# Vitamin B12 deficiency: case report and review of literature

**DOI:** 10.11604/pamj.2021.38.237.20967

**Published:** 2021-03-04

**Authors:** Brahim El Hasbaoui, Nadia Mebrouk, Salahiddine Saghir, Abdelhkim El Yajouri, Rachid Abilkassem, Aomar Agadr

**Affiliations:** 1 Department of Pediatrics, Military Teaching Hospital Mohammed V, Faculty of Medicine and Pharmacy, University Mohammed V, Rabat, Morocco,; 2 Department of Pediatrics, Children's Hospital, Faculty of Medicine and Pharmacy, University Mohammed V, Rabat, Morocco

**Keywords:** Vitamin B12 deficiency, breastfeeding, pallor, psychomotor regression, case report

## Abstract

Vitamin B12 deficiency in early childhood is an important cause of neurodevelopmental delay and regression. Most of these cases occur in exclusively breast-fed infants of deficient mothers. Symptoms and signs of vitamin B12 deficiency appear between the ages of 2 to 12 months and include vomiting, lethargy, failure to thrive, hypotonia, and arrest or regression of developmental skills. Approximately one half of this cases exhibit abnormal movements, variously described as tremors, twitches, chorea, or myoclonus. Urinary concentrations of methylmalonic acid and homocysteine are characteristically elevated in vitamin B12 deficiency. Hyperglycinuria is sometimes present. The early diagnosis and treatment of vitamin B12 deficiency is crucial for significant neurological impairment and long-term prognosis. Treatment with vitamin B12 corrects these metabolic abnormalities very rapidly (within a few days). Vitamin B12 supplementation of pregnant women may prevent neurological and neuroradiological findings of the infants. Because of the importance of vitamin B12 in the development of the foetal and neonatal brain, vegetarian and vegan mothers should be aware of the severe and not fully-reversible damages caused by insufficient nutritional intake of vitamin B12 during pregnancy and lactation. Therefore, efforts should be directed to prevent its deficiency in pregnant and breastfeeding women on vegan diets and their infants. It is also important to take the nutritional history of both infants and their mothers for the early prevention and treatment. Here an interesting case of vitamin B12 deficiency in a 10-month-old boy presented with psychomotor regression, hypotonia and lethargy.

## Introduction

Vitamin B12 deficiency is a rare and treatable cause of failure to thrive and delayed development in infants. In developed countries this deficiency usually occurs in infants who are exclusively breastfed, whose mothers have unrecognized pernicious anemia or are vegetarians, causing low stores of vitamin B12 in the infant at birth and inadequate amounts of the vitamin in the breast milk. Symptoms and signs of vitamin B12 deficiency appear between 2 to 12 months and include vomiting, lethargy, failure to thrive, hypotonia, and arrest or regression of developmental skills. Treatment with vitamin B12 corrects these metabolic abnormalities very rapidly within a few days. We present here a case of vitamin B12 deficiency causing pallor, psychomotor regression, hypotonia and lethargy.

## Patient and observation

We report a 10-month-old boy, presented with history of progressive hypotonia, pallor and failure to thrive. Medical history taken from his mother revealed that he was born at term, weighing 3500g after uncomplicated pregnancy and delivery. No parental consanguinity was observed. He was on exclusively breastfed, and his mother had been following a vegetarian diet for many years. The case showed normal developmental features up to 6 months: smiling at 2 months, controlling his head at 4 months, and starting to roll at 5 months. During the first 6 months of life, his weight, length and psychomotor development were within the normal range. When the child became 6 months old, his parents recognized that their baby stopped gaining weight and became less active. Although brisk reflexes and cranial nerve examination were normal on admission, he was pale, lethargic, generally hypotonic, lacking smiling and failing to follow objects visually, he was unable to sit and was making no attempt to crawl or vocalise. Her weight was below the 3^rd^ percentile. No other abnormal findings were evident on physical examination.

**Figure 1:**
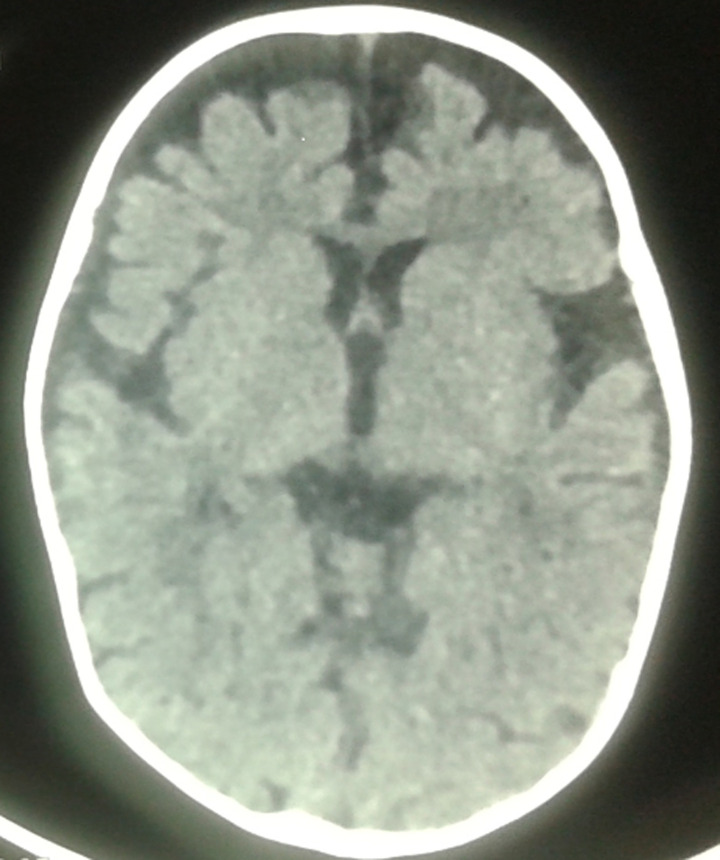
axial proton density-weighted images with delay in myelination in the periventricular white matter with cortical atrophy

Laboratory investigations demonstrated macrocytic anaemia (haemoglobin 6 g/dl, mean corpuscular volume 112fl), hypoalbuminemia (23 mg/l), granulocyte and platelet counts were 5.5x103/mm^3^, and 260x103/mm^3^ respectively, reticulocyte count, red blood cell count, and haematocrit were 6x103/mm^3^, 2.63x106/mm^3^, and 21.3% respectively. The serum cobalamin level was very low 35pg/mL (reference range 211-911 pg/mL) while Serum folate level was normal 13.85 ng/mL (reference range 3.1-20 ng/mL). Iron and ferritin levels, biochemical profile, and urine test results were normal, with normal blood and urine aminoacidography. His mother´s vitamin B12 level was also low at 107 ng/l. Psychomotor regression due to vitamin B12 deficiency was diagnosed through a combination of clinical and laboratory findings, including clinical presentation, increased mean corpuscular volume (macrocytosis) and low vitamin B12 level, while the brain computed tomography (CT) revealed cerebral atrophy with delayed myelination (Figure 1).

The case was given intramuscular injection of 1 mg of cobalamin and displayed a prompt neurological recovery. Three days after the first injection, he was smiling again and was neither lethargic nor hypotonic any longer. Haematologic values improved at the second week of the treatment. His parents were pleased because the case had a great improvement in his mental and motor development. Thus, the communication skills were improved with parents, the appetite became ameliorated, and he could control his head again. He started walking when he was 18 months old. Control cranial magnetic resonance imaging (MRI) performed three months after the initiation of therapy demonstrated recovery of cerebral atrophy, subarachnoid space was in normal range. With his neurologic improvement and normal haematologic values, cobalamin therapy was discontinued at the age of 18 months.

## Discussion

Vitamin B12 is not synthesized in human beings, and the only dietary source is animal products. During the antenatal period, vitamin B12 is actively transferred to the fetus through the placenta. An infant of vitamin B12 replete mother has hepatic stores of 25 mg of vitamin B12 at birth and obtains 0.25 mg/d from breast milk, if exclusively breastfed. In deficient mothers, hepatic stores of infants at birth are much lower with more pronounced effect on breast milk content [1]. Strict vegetarian mothers are at greatest risk of vitamin B12 deficiency, with lacto-ovo vegetarians being at a higher risk than omnivores [2]. In all recent case series reporting vitamin B12 deficiency with neurologic symptoms in infants, maternal dietary deficiency has been the predominant cause [3, 4]. Other important causes of vitamin B12 deficiency in mothers are pernicious anemia, gastric bypass, and holotranscobalamin deficiency [5]. Although many of the metabolic disturbances resulting from vitamin B12 deficiency are understood, the pathogenesis of the nervous system abnormalities is unknown. Several cofactors, derived from vitamin B12, are necessary for the conversion of homocysteine to methionine and methylmalonyl-CoA to succininyl-CoA and when these cofactors are unavailable, abnormal levels of homocysteine are found in the blood and excessive methylmalonic acid is excreted in the urine. Treatment with vitamin B12 corrects these metabolic abnormalities very rapidly within a few days.

Vitamin B12 is essential for development of the central nervous system. Nutritional vitamin B12 deficiency is a treatable cause of failure to thrive and delayed neurodevelopment in infants. Nutritional vitamin B12 deficiency in infancy is common in turkey and has been reported in previous studies [6]. In underdeveloped or developing countries, the deficiency usually occurs in infants who are exclusively breastfed, with mothers who are vegetarians, causing low stores of vitamin B12 in the infant at birth and inadequate amounts of vitamin in the breast milk [7]. Long-term deficiency of the vitamin B12 may cause demyelination of the brain, but the exact mechanism of vitamin B12 in the metabolism of the nervous system is not fully understood [8, 9]. Although the role of vitamin B12 in neuromotor development is well-known in infants, there is limited information and only a few cases report available concerning. Deficiency of cobalamin, the cobalt-containing complex common to Bl2, leads to two major clinical syndromes. One is expressed clinically, not only as megaloblastic hematopoiesis but as defective proliferation of all rapidly dividing cells, with sequelae such as glossitis, partial villous atrophy, and hypospermia attributable to impaired Deoxyribonucleic acid (DNA) synthesis. On the other side neurological damage is thought to occur in the first 6 months of life in infants, when myelination of the brain is very active. Lack of vitamin B12 in the maternal diet during pregnancy has been shown to cause severe retardation of myelination in the nervous system [8]. Vitamin B12 deficiency may lead to impaired synthesis of ethanolamine, phospholipids, and sphingomyelin resulting in altered myelin integrity with deficient long fiber tract function and axonal neuropathy [10] that causing progressive lethargy and delayed development with subsequent, neuromotor developmental delay, nutritional disorders, convulsions, hypotonia, involuntary movements, hypothermia, and coma [11].

The various neuroradiological findings may be observed on cranial imaging in infants with vitamin B12 deficiency. Cortical atrophy, thinning of the corpus callosum, structural abnormalities and retardation in myelination are the most frequent neuroradiological findings in vitamin B12 deficiency [12, 13]. Extensive lesions in the brain itself have only been found in a few cases showing small perivascular areas of demyelination within the white matter [8]. The long-term prognosis depends on overall duration of deficiency and severity of symptoms rather than serum levels of vitamin B12 or haemoglobin values on admission. It seems that infants diagnosed and treated before one year of age have more favorable neurological outcome than those treated at a later period, after correction of B12 deficiency, these children show rapid improvement in their general condition with increased activity, interest in surroundings, and increased appetite. The United Nations Children´s Fund (UNICEF) recognizes that dietary deficiency of micronutrients, including vitamin B12, affects millions of people throughout the world and compels one-third of the world´s population to live below their physical and mental potential [14]. A World Health Organization technical consultation on vitamin B12 and folate deficiencies (2008) concluded that vitamin B12 has a clear impact on child development and affects the cognitive scores of school-aged children. Food fortification and supplementation of the target population groups has been suggested as an optimal approach to this problem [15].

## Conclusion

Vitamin B12 is important for development of brain. Severe and various neurological and neuroradiological findings may be seen in infants due to vitamin B12 deficiency. It should be considered in infants with hypotonia or neurodevelopmental retardation and with neuroradiological findings such as thinning of the corpus callosum, cortical atrophy and retardation in myelination. Early recognition of these infants is important as this condition is partially reversible. Because of the importance of vitamin B12 in the development of the foetal and neonatal brain, vegetarian and vegan mothers should be aware of the severe and not fully-reversible damages caused by insufficient nutritional intake of vitamin B12 during pregnancy and lactation. Therefore, efforts should be directed to prevent its deficiency in pregnant and breastfeeding women on vegan diets and their infants.

## References

[1] Casella EB, Valente M, de Navarro JM, Kok F (2005). Vitamin B12 deficiency in infancy as a cause of developmental regression. Brain Dev.

[2] Majchrzak D, Singer I, Manner M, Rust P, Genser D, Wagner KH (2006). B-vitamin status and concentrations of homocysteine in Austrian Omnivores, vegetarians and vegans. Ann Nutr Metab.

[3] Oya Halicioglu, Sezin Asik Akman, Sumer Sutcuoglu, Berna Atabay, Meral Turker, Sinem Akbay (2010). Neurological findings of nutritional vitamin B12 deficiency in children. Turk J Pediatr.

[4] Oya Halicioglu, Sezin Asik Akman, Sumer Sutcuoglu, Berna Atabay, Meral Turker, Sinem Akbay (2011). Nutritional B12 deficiency in infants of vitamin B12-deficient mothers. Int J Vitam Nutr Res.

[5] Banka S, Roberts R, Plews D, Newman WG (2010). Early diagnosis and treatment of cobalamin deficiency of infancy owing to occult maternal pernicious anemia. J Pediatr Hematol Oncol.

[6] Taskesen M, Okur N, Katar S, Okur N, Soker M (2009). Nutritional megaloblastic anemia during childhood: demographical, clinical and laboratory features of 134 patients from southeastern part of Turkey, e-SPEN. Eur e-Journal Clin Nutr Metab.

[7] Chalouhi C, Faesch S, Anthoine-Milhomme MC, Yvonne Fulla (2008). Neurological consequences of vitamin B12 deficiency and its treatment. Pediatr Emerg Care.

[8] Lövblad K, Ramelli G, Remonda L, Nirkko AC, Ozdoba C, Schroth G (1997). Retardation of myelination due to dietary vitamin B12 deficiency: cranial MRI findings. Pediatr Radiol.

[9] Healton EB, Savage DG, Brust JCM, Garrett TJ, Lindenbaum J (1991). Neurologic aspects of cobalamin deficieny. Medicine (Baltimore).

[10] Codazzi D, Sala F, Parini R, Langer M (2005). Coma and respiratory failure in a child with severe vitamin B 12 deficiency. Pediatr Crit Care Med.

[11] Wighton MC, Manson JI, Speed I, Robertson E, Chapman E (1979). Brain damage in infancy and dietary vitamin B12 deficiency. Med J Aust.

[12] Stollhoff K, Schulte FJ (1987). Vitamin B12 and brain development. Eur J Pediatr.

[13] Von Schenck U, Gotze CB, Koletzko B (1997). Persistence of neurological damage induced by dietary vitamin B12 deficiency in infancy. Arch Dis Child.

[14] United Nations of International Children's Emergency Fund (UNICEF) (2004). Vitamin and mineral deficiencies: a global progress report.

[15] de Benoist B (2008). Conclusions of a WHO Technical Consultation on folate and vitamin B12 deficiencies. Food Nutr Bull.

